# Pediatric Knee Injury: A Unique Case of Intra-articular Osteochondral Fracture Following Penetrating Trauma

**DOI:** 10.7759/cureus.53236

**Published:** 2024-01-30

**Authors:** Alexandros E Koskiniotis, Nikolaos Stefanou, Nikolaos Metaxiotis, Vasileios Amprazis, Sokratis Varitimidis

**Affiliations:** 1 Department of Orthopaedic Surgery and Musculoskeletal Trauma, University Hospital of Larissa, Larissa, GRC

**Keywords:** open fracture, traumatic arthrotomy, osteochondral fracture, intra-articular fracture, penetrating trauma

## Abstract

Penetrating injuries to the musculoskeletal system pose common challenges for orthopedic surgeons in emergency departments (EDs). The complexity escalates when a joint is affected, increasing the risk of severe complications such as infection and post-traumatic arthritis. Given the potential importance of these injuries, early diagnosis and a meticulous treatment plan are crucial. In this paper, we present a unique case of penetrating trauma, resulting in an intra-articular defect on the lateral femoral condyle of an adolescent girl. This case underscores the importance of tailored interventions in managing complex musculoskeletal injuries.

## Introduction

Penetrating trauma to the musculoskeletal system represents a common challenge encountered by orthopedic surgeons in emergency departments (EDs). Such injuries can result from various causes, ranging from simple falls to direct blows by sharp objects or even gunshot wounds. However, when a penetrating injury involves a joint, the complexity of the case significantly increases, elevating the risk of infection or even secondary traumatic arthritis when there is a bone injury as well [1]. During adolescence, the biomechanical strength of the immature osteochondral junction is lower than that of adult patients [2]. Such injuries demand immediate attention and a multidisciplinary approach that includes orthopedic surgeons, radiologists, and trauma specialists. Early diagnosis, thorough evaluation through advanced imaging techniques, and precise surgical intervention are essential for achieving optimal outcomes. In this study, we present a rare case of a penetrating knee injury in a pediatric patient involving an osteochondral fragment from the lateral femoral condyle. Osteochondral fractures of the lateral condyle of the femur are rare but can frequently cause significant knee joint pain and joint degeneration [3].

## Case presentation

**Figure 1:**
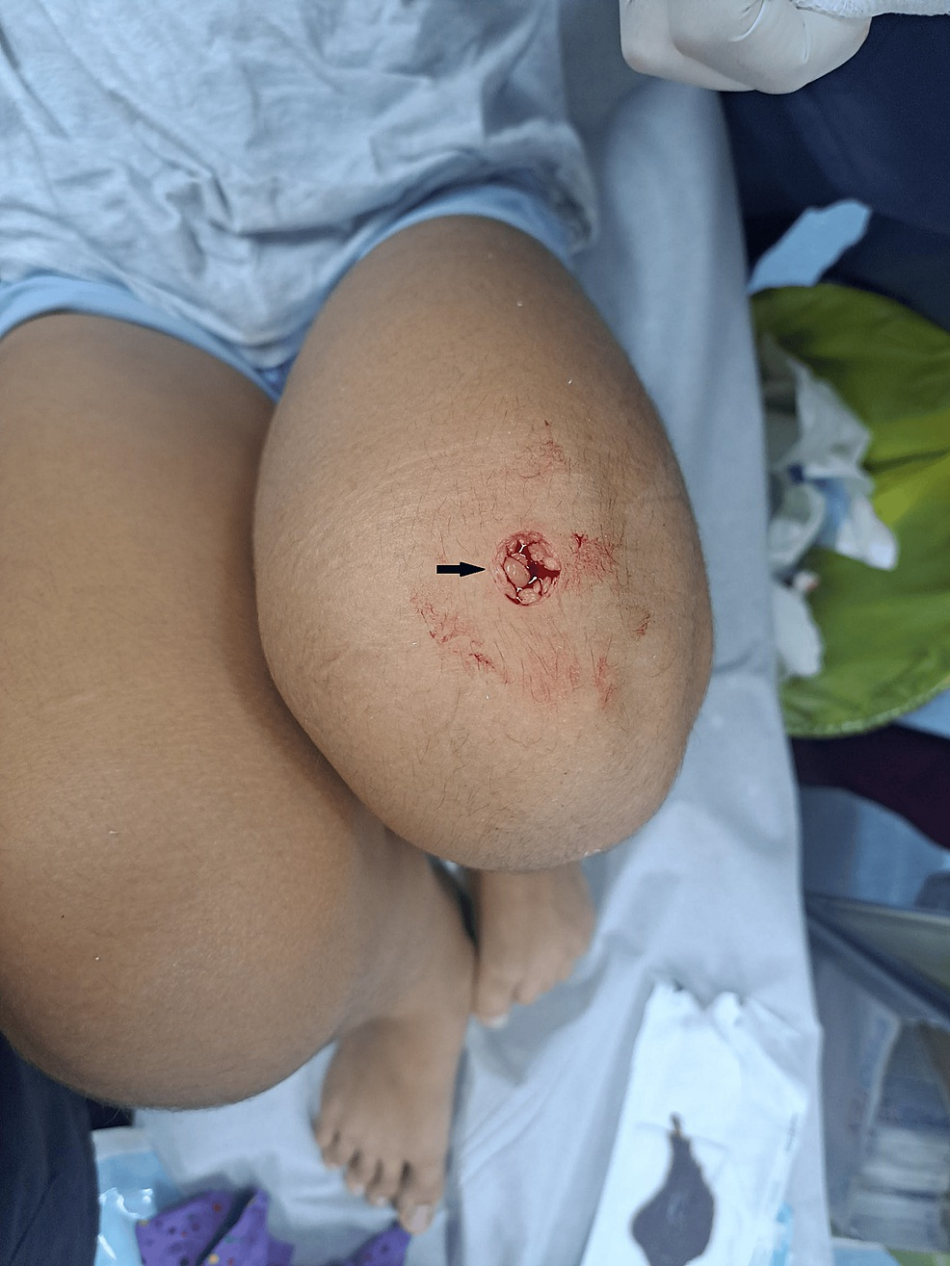
A circular-shaped wound just above the patella, caused by a direct blow from the door knob.

A 13-year-old girl, accompanied by her mother, presented to the ED after falling from height, resulting in a direct impact to her left knee from a tube-shaped door knob. The patient exhibited a circular-shaped wound just above and laterally to the patella (Figure 1).

**Figure 2:**
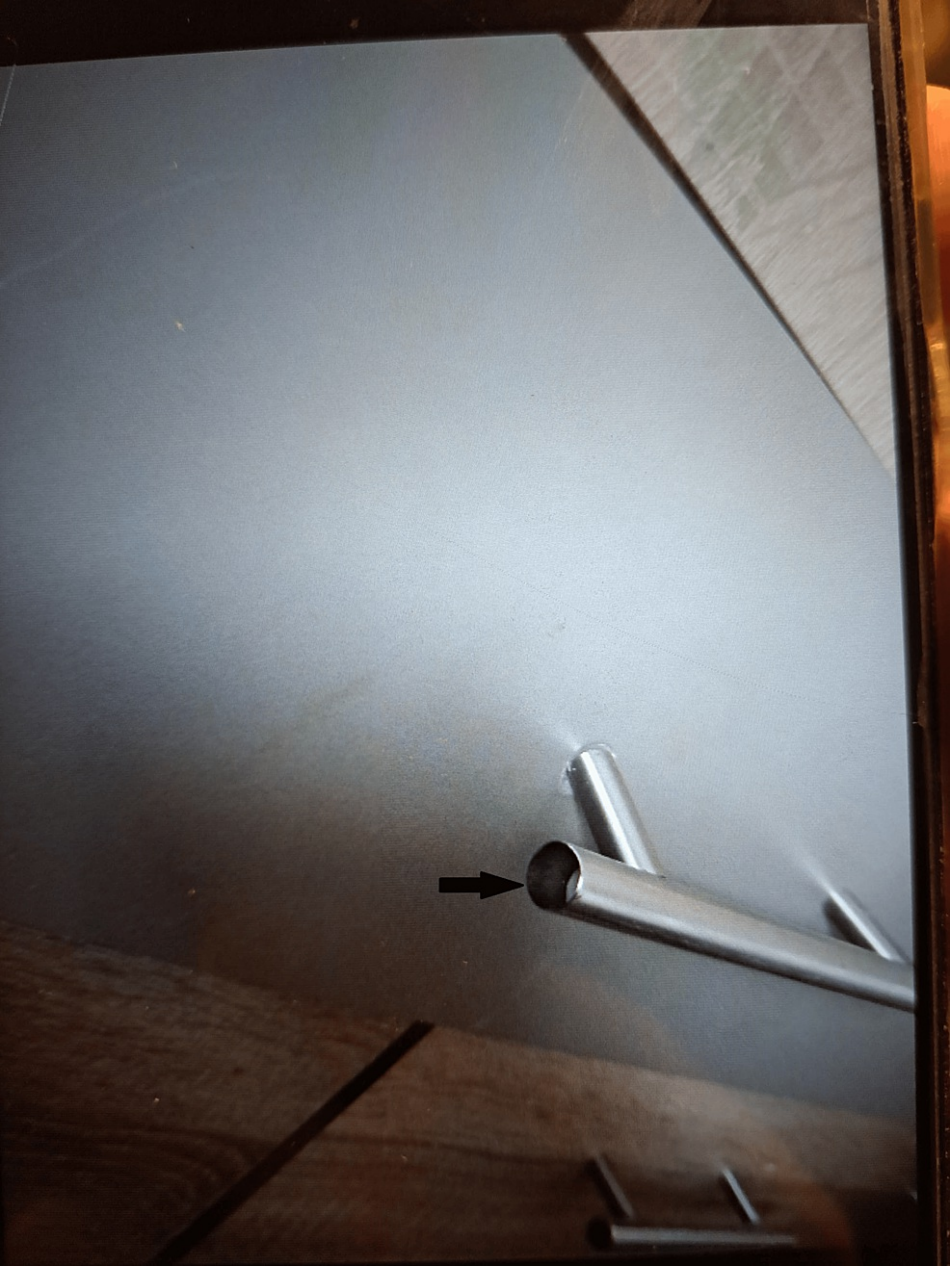
The bone fragment is barely visible inside the door knob itself.

She experienced significant pain, rendering her unable to walk. Physical examination revealed swelling of the left knee, local tenderness, and limited flexion and extension of the left knee joint with range of motion (ROM): Flexion/Extension 90°/0°. Her mother brought with her an arrow-shaped fragment of bone collected from the door knob itself (Figure 2).

**Figure 3:**
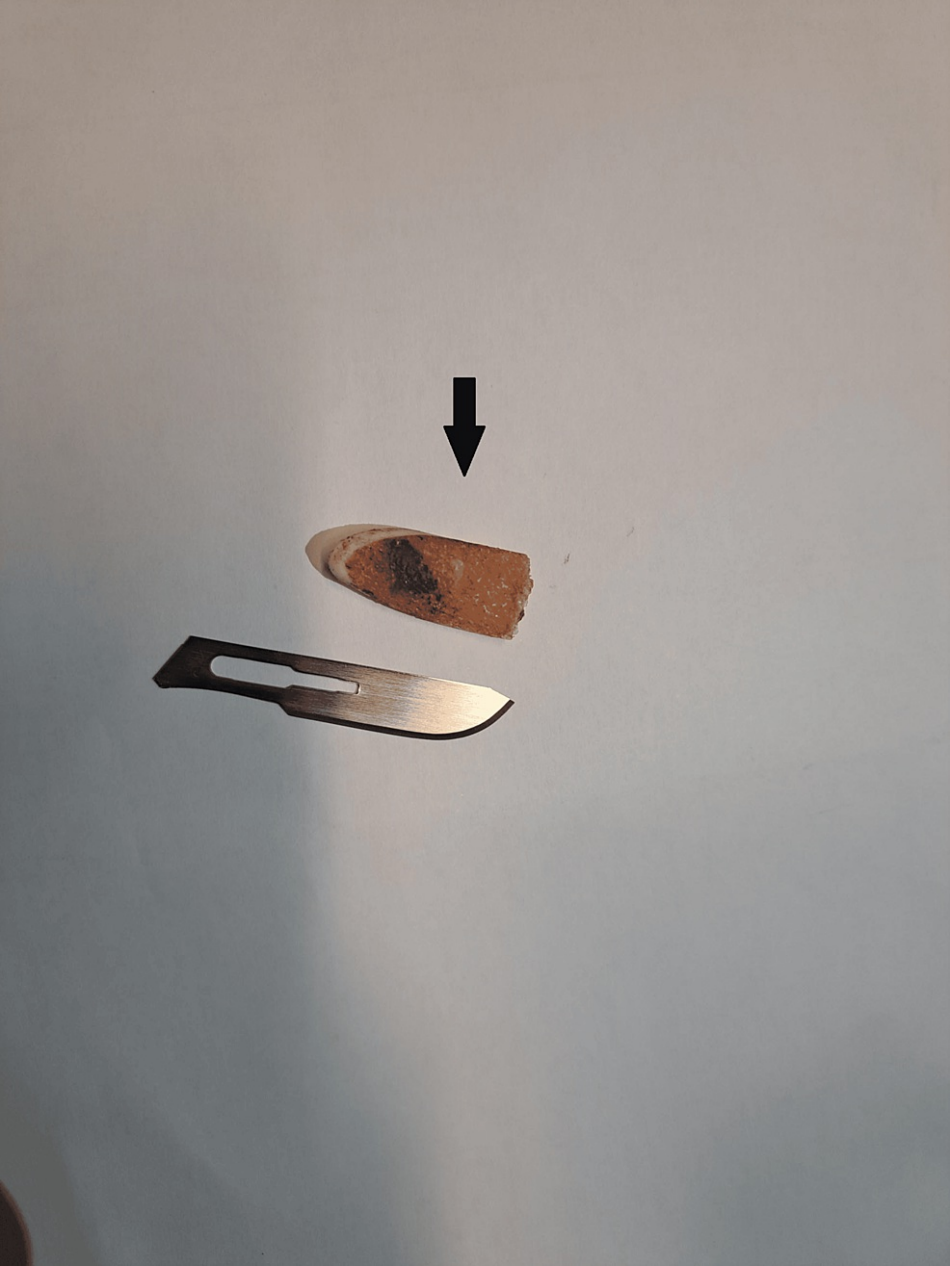
The arrow-shaped bone fragment from the patient’s left knee, approximately 2 cm in length.

The fragment measured, in its largest dimension, approximately 2 cm, making it challenging to ascertain its origin, specifically whether it had an articular connection (Figure 3).

**Figure 4:**
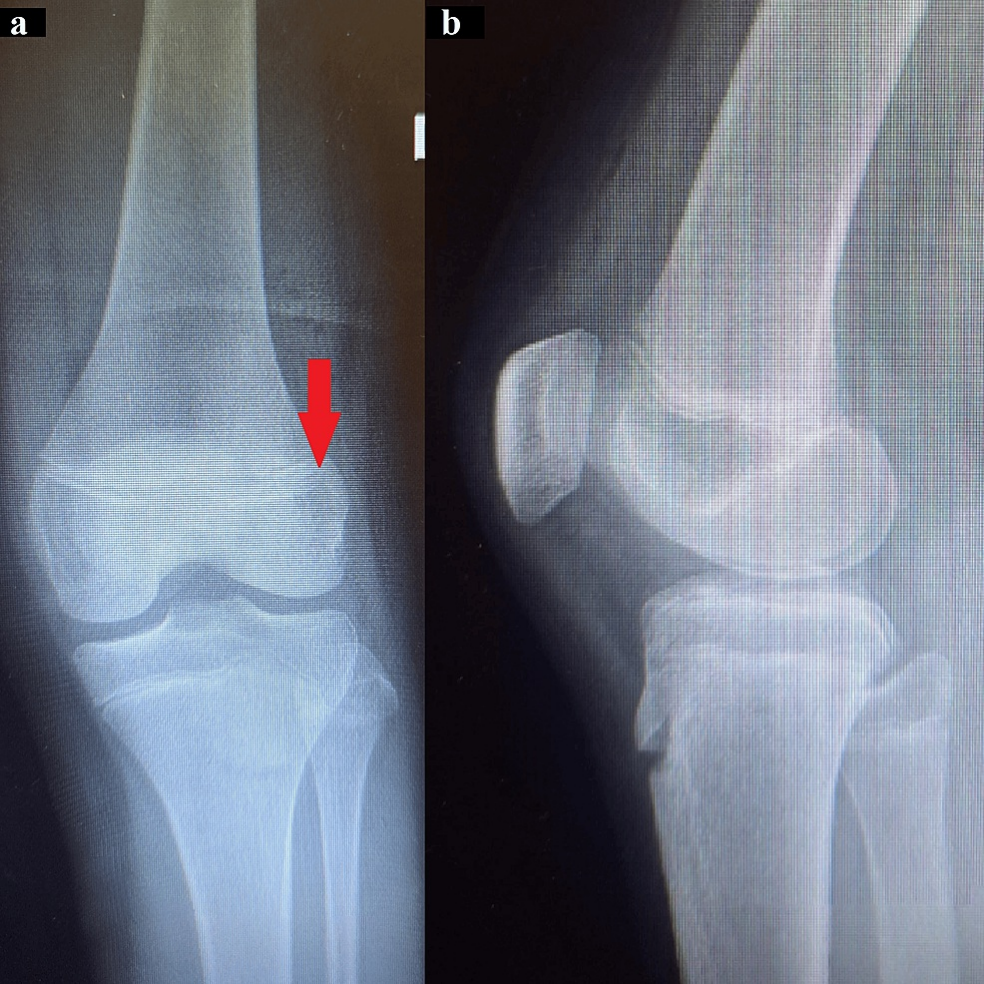
The X-rays were inconclusive, without a clear sign of an intra-articular lesion from the lateral femoral condyle. (a) Anteroposterior X-ray of the patient's affected knee shows the lateral femoral condyle appearing normal. (b) Lateral X-ray of the patient's injured knee reveals no evidence of bone fragments.

After receiving thorough wound care and antibiotic prophylaxis in the ED, including intravenous cefoxitin and amikacin at dosages according to the guidelines for her age, inconclusive findings were observed from an X-ray of the affected knee (Figure 4).

**Figure 5:**
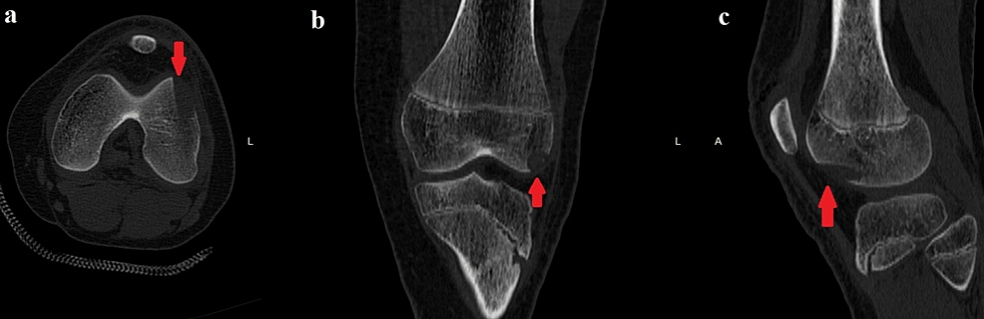
CT scan of the patient’s left knee. The intra-articular lesion is visible on the lateral femoral condyle: (a) axial view; (b) coronal view; and (c) sagittal view. CT, computed tomography

A CT scan was performed to determine the fragment’s anatomical association with the joint. The CT scan revealed a lesion in the lateral femoral condyle just below the physis (Figure 5).

**Figure 6:**
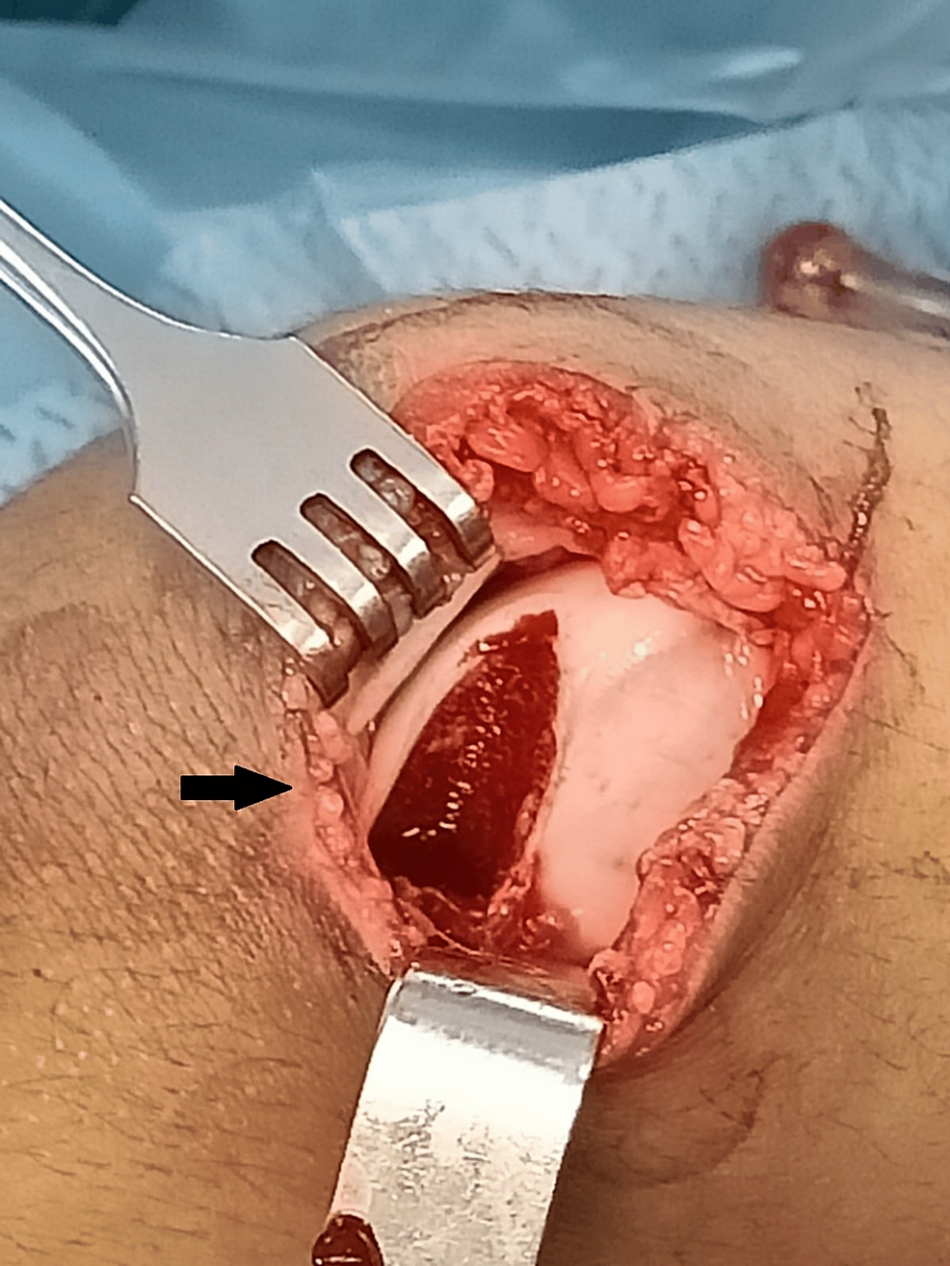
Operative view from the intra-articular missing fragment of the knee joint.

The girl was promptly admitted to the orthopedic department and subsequently to the operating room, where she underwent open debridement and lavage of the knee joint (Figure 6).

**Figure 7:**
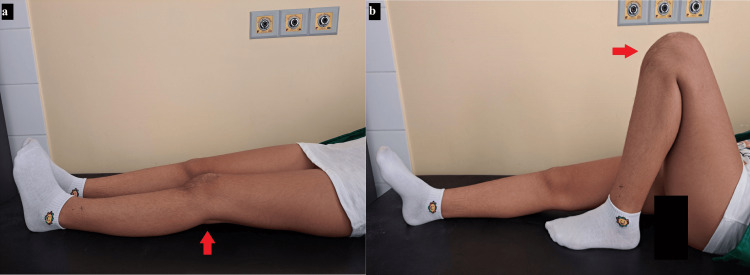
The patient regained her normal range of motion and functionality over the course of six weeks. (a) The patient's affected limb in full extension; (b) full flexion of the operated knee without discomfort.

Microfractures were also performed to promote bone growth in the area of the lesion. After two days in the orthopedic department following the same antibiotic prophylaxis protocol, the patient returned to her home with guidelines for partial weight-bearing and active ROM exercises. Over the course of six weeks of rehabilitation, she regained her normal ROM and functionality in the affected limb (Figure 7).

The Modified Hospital for Special Surgery (Modified HSS) knee score yielded excellent results with 88 points, and the patient experienced no discomfort in the knee joint during daily activities, with an ROM of flexion/extension at 145°/0°. Four months postoperatively, the patient was allowed to carry out normal physical exercise, including contact sports.

## Discussion

According to the published literature, open periarticular injuries are most commonly encountered in the knee when compared to other joints of the lower and upper extremities. These injuries also tend to affect males more frequently than females [4-6]. Traumatic arthrotomies, such as the one seen in our case, are often caused by high-impact events, including traffic accidents, gunshot wounds, and stab injuries. Considering that high-energy trauma is the usual cause of these injuries, it is crucial to rule out any neurovascular injury that might be limb-threatening during our first assessment of the patient. If the perforation of the injured limb is in doubt after a thorough clinical examination, including the evaluation of hard and soft signs, we should promptly proceed to an emergency CT angiography for a comprehensive assessment. Finally, we should not forget that the neurovascular status of a limb might also be at risk due to a possible compartment syndrome for the next 24 to 48 hours following the traumatic event [7].

Pediatric fractures affecting the metaphyseal and epiphyseal regions are commonly classified using the Salter-Harris classification, which was first introduced in 1963. This classification system defines five fundamental fracture types [8]. Over the years, certain modifications and additions have been proposed by different authors to further enhance its specificity. Ogden in 1983 [9] added three more types in the S-H classification, with type VII representing an isolated epiphyseal fracture, unrelated to the physis. These types of injuries are primarily located at the malleoli and within the distal humerus or distal femur, manifesting as osteochondral fractures. In our case, the fracture pattern in this pediatric patient could be categorized as S-H Ogden VII fracture.

An osteochondral fracture is characterized by damage to both the articular cartilage and the underlying subchondral bone. Typically associated with acute traumatic incidents, these fractures are occasionally overlooked or misdiagnosed during initial patient evaluations. Such oversights can result in chronic lesions due to the production of type I collagen in the form of fibrocartilage and, ultimately, in post-traumatic osteoarthritis [10]. While a plain X-ray is the most straightforward method to identify an osteochondral fracture, there are instances where this type of fracture goes undetected through conventional radiography [11]. For diagnosing osteochondral lesions, advanced imaging methods such as CT and MRI scans may be necessary. Finally, treatment approaches vary: non-operative methods are suitable for fragments <1 cm^2^, while arthroscopy can address cases with loose bodies inside the joint. Larger fragments (>1 cm^2^) may require open reduction and internal fixation (ORIF) in acute lesions or an osteochondral autograft/allograft. Additionally, for lesions confined to the articular joint, microfractures represent a viable option for managing larger defects [10]. After surgical management, the patient is typically advised to remain non-weight-bearing on the affected side for approximately six weeks. Early ROM exercises are encouraged during this period, especially when the fracture fixation is stable. Follow-up radiographs postoperatively are essential to ensure the integrity of the joint surface. However, for a comprehensive assessment of the injured cartilage, an MRI scan is necessary during the follow-up period.

A literature review regarding traumatic arthrotomy and its possible complications was published in 2019 by Brubacher et al. [1]. In this paper, the authors discussed the current diagnostic approaches for periarticular wounds like the saline load test and the role of the CT scan. It also explored current treatment protocols aimed at preventing post-traumatic infections and traumatic arthritis. It seems that surgical irrigation and debridement in combination with intravenous antibiotic treatment remains the gold standard method for treating cases of traumatic arthrotomy and minimizing complication rates. However, it's worth noting that non-operative management may yield comparable results in cases of smaller open intra-articular injuries, offering the potential to reduce overall hospitalization costs for patients [12]. In the end, the authors conclude that our current knowledge in this field is limited by outdated, low-evidence studies. To provide more precise guidance for treatment selection, further research is essential, particularly through the analysis of larger databases. Such investigations can help tailor treatment decisions based on injury characteristics, such as size, degree of contamination, and associated intra-articular fractures.

Before the widespread availability of CT scans in EDs worldwide, the saline load test was a common method used to assess whether a periarticular injury extended into the joint. First described by Patzakis et al. in 1975 [4], the saline load test involves injecting sterile normal saline into the affected joint space, away from the periarticular wound. If leakage of the saline is observed through the wound, the clinical test is considered positive. Over the years, various researchers have explored the test's effectiveness, not only for knee injuries but also for injuries involving other joints, with clinical and cadaveric studies examining also the proper amount of saline that needs to be injected to have a positive result [13-15]. According to these studies, the load saline test can achieve sensitivity up to 95%, making it a quite useful tool in diagnosing a potential traumatic arthroscopy.

The significance of the CT scan in recognizing intra-articular lesions after a penetrating trauma has been studied by various authors according to published literature. For instance, Konda et al. studied 78 patients who presented in the ED with deep knee wounds, raising suspicion of possible traumatic arthrotomy. According to the results of the study, CT scans successfully detected nine additional intra-articular fractures that had gone unnoticed on conventional X-rays. It seems that CT emerges as an invaluable tool for physicians, not only in identifying open fractures of the joint but also in diagnosing traumatic arthroscopy through the detection of air within the affected joint. Given its proven efficacy, there is a compelling argument for considering the inclusion of CT scans as a routine component in the diagnostic algorithm for patients presenting in the ED with deep periarticular injuries [16].

Microfracture is a marrow-stimulating technique designed to facilitate the formation of fibrocartilage tissue in response to an intra-articular chondral lesion. This method involves creating perforations through the subchondral region, allowing the influx of blood and marrow elements into the cartilage defect area, forming a clot rich in stem cells and growth factors. Over time, this clot transforms into fibrocartilage [17]. Despite fibrous cartilage not matching the strength of normal hyaline cartilage found in joints, microfracture is a reliable procedure known to provide pain relief, even in professional athletes [18]. The size of the lesion is also not a contraindication for microfracture [19]. In our case, the patient's bone fragment, contaminated by the injury, could not be used. The microfracture technique was evaluated as the optimal choice, considering the non-weight-bearing anatomic location of the lesion and the heightened risk of complications associated with using a different area for an osteochondral autograft.

## Conclusions

In conclusion, penetrating musculoskeletal trauma, particularly injuries involving joints, presents a complex and challenging clinical scenario demanding immediate measures by an orthopedic surgeon. Understanding the precise details of how the injury occurred is crucial because in the hustle of the emergency room, particularly with smaller wounds near joints, serious intra-articular injuries might be overlooked. Finally, higher clinical suspicion and careful assessment of the patient are recommended in all injuries surrounding nearby joints, as prompt and accurate treatment can significantly impact a patient's quality of life and functional recovery.
